# Subretinal Amniotic Membrane Transplantation in a Porcine Model of Retinal Hole

**DOI:** 10.1167/iovs.65.13.52

**Published:** 2024-11-25

**Authors:** Madeline E. Olufsen, Jens Hannibal, Nina B. Sørensen, Anders T. Christiansen, Ulrik C. Christensen, Grazia Pertile, David H. Steel, Steffen Heegaard, Jens F. Kiilgaard

**Affiliations:** 1 Department of Ophthalmology, Rigshospitalet, University of Copenhagen, Copenhagen, Denmark; 2 Faculty of Health and Medical Sciences, Institute of Clinical Medicine, University of Copenhagen, Copenhagen, Denmark; 3 Department of Clinical Biochemistry, Faculty of Health Sciences, Bispebjerg and Frederiksberg Hospital, University of Copenhagen, Copenhagen, Denmark; 4 IRCCS Sacro Cuore Don Calabria Hospital, Verona, Italy; 5 Bioscience Institute, Newcastle University, Newcastle Upon Tyne, United Kingdom; 6 Department of Pathology, Rigshospitalet, University of Copenhagen, Copenhagen, Denmark

**Keywords:** healing process, pig, subretinal surgery, immunohistochemistry, amniotic membrane

## Abstract

**Purpose:**

To investigate the histopathological changes following subretinal amniotic membrane transplantation in an in vivo porcine model of retinal holes.

**Methods:**

Left eyes of 12 Danish Landrace pigs were vitrectomized under full anesthesia. A subretinal bleb was created before excising a retinal hole (1154–2934 µm) using a 23-gauge vitrector. The pigs underwent transplantation of human freeze-dried amniotic membrane into the subretinal space, with no tamponade applied. Optical coherence tomography and color fundus photography were performed just after surgery and at 2 and 4 weeks post-surgery. At the end of follow-up, the eyes were enucleated for hematoxylin and eosin staining and fluorescence immunohistochemistry, using antibodies against retinal glial cells and inner retinal neurons.

**Results:**

The amniotic membrane sheets facilitated hole closure by gliosis and centripetal migration of the edges of the hole. Immunohistochemical examination showed that the cells within the closed hole expressed anti–glial fibrillary acidic protein (GFAP) and anti-S100B, but not anti–glutamine synthetase (GS), suggesting that astrocytes were the predominant glial cells involved in hole closure. Gliosis was observed between the amniotic membrane sheet and the overlying photoreceptors of the surrounding retina. Morphological restoration of the retinal layers within the closed retinal hole was not observed.

**Conclusions:**

The amniotic membrane acted as a stimulator for retinal hole closure by inducing glial cell proliferation and providing a scaffold for the centripetal migration of the edges of the hole. No morphological restoration was observed.

Full-thickness macular holes (MHs) refractory to pars plana vitrectomy and internal limiting membrane (ILM) peeling present a great challenge. Recently, various new surgical approaches to attempt MH closure have been introduced. Several of the new techniques place a tissue graft into or above the MH. The grafts include an inverted or free autologous ILM flap,^1^^–^^3^ a posterior or anterior autologous lens capsular flap,^4^ autologous neurosensory retinal flap transplantation (ART),^5^^,^^6^ or a human amniotic membrane (hAM).^7^ The rationale of using tissue grafts is that they might induce cell proliferation, serve as a scaffold for retinal regeneration, or even, regarding ART, have the potential to become sensory functional by synaptic contact with the edges of the MH.^5^^,^^7^^–^^9^

Transplantation of a hAM into the subretinal space was first introduced by Rizzo et al.^7^ for patients with retinal breaks and recurrent MHs. Since then, several other studies have followed, including closure of large, myopic and refractory MHs.^10^^–^^14^ Follow-up examinations have shown high anatomical closure rates and visual acuity improvement.^7^^,^^10^^–^^13^ In the subretinal space, the amniotic membrane (AM) prevents contact between the vitreous and the retinal pigment epithelium (RPE), stimulates proliferation and differentiation of the RPE, and might act as a scaffold for retinal ingrowth and differentiation of retinal layers.^7^^,^^12^^,^^15^ Furthermore, the AM may, similarly to an ILM flap, induce glial cell proliferation, resulting in filling of the MH with proliferating cells that may provoke gliosis.^1^^,^^16^

The AM has shown reconstructive and regenerative capacities in the treatment of ocular surface abnormalities, attributed to the production of growth factors and expression of adhesion molecules in the amniotic stroma.^17^^,^^18^ This regenerative potential has earlier been demonstrated in retinal structures, as well. Capeáns et al.^19^ cultured human RPE cells in vitro over a hAM sheet and demonstrated that hAM serves as a suitable substrate for growth and proliferation of RPE cells. Kiilgaard et al.^20^^,^^21^ later showed that an AM sheet transplanted into the subretinal space in pigs that had undergone central RPE debridement became covered with the host's RPE and that the induced proliferation of RPE cells occurred in the periphery retina. This regenerative potential of the AM could explain the visual gain observed during follow-up examinations in patients with treated MHs, but histological evaluations are necessary to conclude this.

Furthermore, the cellular mechanism by which the AM transplant promotes hole closure is as yet unknown. We hypothesized that AM promotes retinal hole closure by stimulating gliosis or the regeneration of retinal cells. To study these two possibilities, we therefore evaluated the histopathological changes after subretinal AM transplantation in an in vivo porcine model of retinal holes.

## Materials and Methods

**Figure 1.**
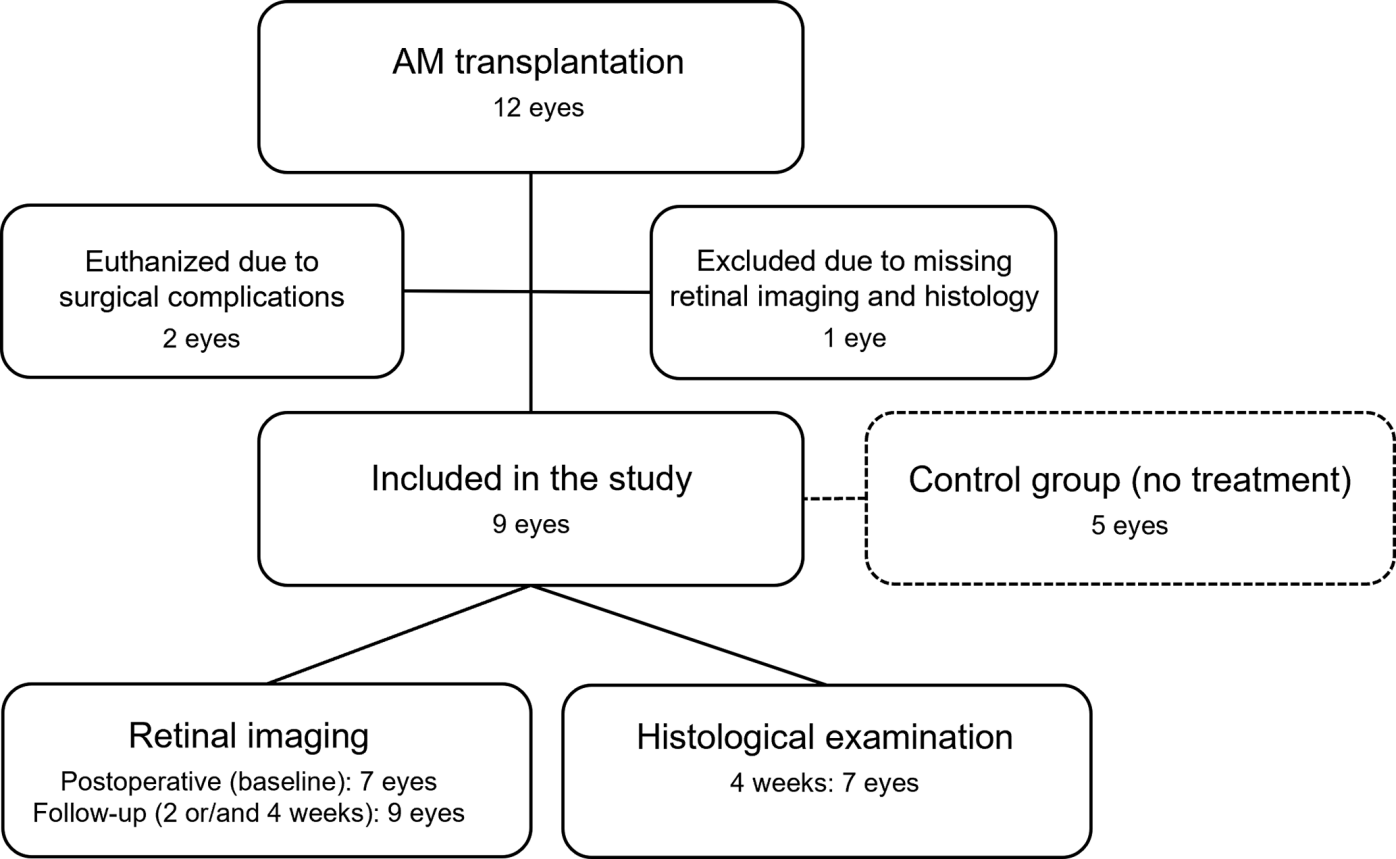
Flow chart of the included eyes. Twelve eyes underwent AM transplantation, and nine eyes were included in the study. Immediate postoperative (baseline) imaging was only available in seven eyes due to air tamponade in the remaining eyes. Retinal imaging was performed on all eyes during follow-up. Histological examination of the seven eyes that achieved hole closure was performed. The control group consisted of five eyes that underwent a similar procedure, wherein the holes were left untreated and closed spontaneously.

The left eyes of 12 3-month-old female Danish Landrace domestic pigs with an average weight of 25 kg underwent surgery (Fig. 1). Two pigs were euthanized and excluded from the study at day 0 due to surgical complications—namely, iatrogenic cataract and severe retinal hemorrhage due to a peripheral retinal tear. One pig with a retinal hole of 1900 µm in basal diameter and dislocated AM was excluded, as severe retinal detachment (RD) made follow-up examinations and histological preparation irrelevant. As control, we used five eyes from a previous study that achieved spontaneous hole closure within the same porcine model.^22^

The retinal holes were established within the “visual streak” of the porcine retina, which is an area with a high density of photoreceptors and ganglion cells comparable to the human macula.^23^^,^^24^ Animal treatment was in accordance with the ARVO Statement for the Use of Animals in Ophthalmic and Vision Research and was supervised by a veterinarian. The research protocol was approved by the Danish Animal Experiment Committee (#2019-15-0201-01652).

### Anesthesia and Surgical Procedure

All surgeries and follow-up examinations were performed under full anesthesia, and the pupil was dilated as previously reported by our group.^25^ Four sclerotomies were performed, at 11, 2, 7, and 5 o'clock, 1 mm posterior to the corneal limbus. After securing an infusion line inferiorly with balanced salt solution (BSS; BSS PLUS Irrigating Solution; Alcon, Geneva, Switzerland), a vitrectomy including separation and removal of the posterior hyaloid was performed using a 23-gauge vitrectomy probe (Constellation 23G ULTRAVIT Vit Probe 7500CPM; Alcon). A subretinal bleb was created by injecting BSS into the subretinal space through a 38-gauge (0.12 mm) cannula (PolyTip; MedOne, Sandy, UT, USA), and subsequently a retinal hole of approximately 1 to 3000 µm was cut with the vitrectomy probe.

Remnants of cryopreserved human AM (NuVision Biotherapies, Nottingham, UK), surplus after corneal surgery in human patients, were used for the study (Danish Research Ethics Committee waived requirements of informed consent from the donors). The AM sheet was placed in the subretinal space below the edges of the retinal hole using 23-gauge ILM forceps (Revolution DSP 23G ILM Forceps; Alcon). The retinal hole was cut to be approximately 0.5 mm smaller than the prepared AM sheet. The sheet was folded with the sticky chorion layer facing inward, which made it easier to maneuver it inside the eye. No tamponade was used in the majority of the cases, except in two eyes where fluid–air exchange was performed; the pigs’ vigorous head movements during recovery from anesthesia raised concerns that the air bubble might cause further harm. Sclera and conjunctiva were sutured with Vicryl 7-0 (Ethicon, New Brunswick, NJ, USA), and chloramphenicol ointment (Kloramfenikol “DAK”; Nycomed, Roskilde, Denmark) was applied in the inferior fornix at the end of the procedure.

### Follow-Up Examinations

Prior to surgery, immediately after and 2 and/or 4 weeks after surgery, the animals were examined with color fundus photography (CFP; ZEISS FF 450; Carl Zeiss Microscopy, Jena, Germany) and optical coherence tomography (OCT; SPECTRALIS OCT; Heidelberg Engineering, Heidelberg, Germany). Retinal hole diameters just after surgery and at follow-up examinations were measured on OCT B-scan as the maximal distance between the outer nuclear layers on both sides of the hole. In the closed holes, where normal stratification was lost, the distance between preserved retinal layering was measured at the outer nuclear layer level. This distance was classified as a retinal plug, as earlier reported by our group.^22^ At the end of follow-up, the pigs were euthanized with 1-mL/kg pentobarbital, 200-mg/mL lidocaine hydrochloride, and 20-mg/mL intravenously (Glostrup Apotek, Glostrup, Denmark), and the left eyes were enucleated.

### Data Analysis

The sizes of both the basal hole and the retinal plug were analyzed using R 4.1.3 (R Foundation for Statistical Computing, Vienna, Austria). Movements of the major retinal vessels were assessed by using the Heidelberg OCT follow-up function, which allowed us to compare baseline images (reference image) with follow-up images at the exact same spot. Inability to use the follow-up function indicated that the vessels had moved. To illustrate vessel movement, baseline and follow-up fundus images were aligned in Photoshop (Adobe, San Jose, CA, USA). All histology was thoroughly examined by one assessor, and the areas of interest were evaluated and discussed within the research group.

### Histological Examinations

Following enucleation, the eyes were immediately fixed in 4% paraformaldehyde. The area containing the optic disc and the retinal hole was identified, isolated, orientated, and embedded in paraffin according to standard procedures. Five sections of 5 µm were cut every 50 µm through the lesion, and every fifth of these sections was stained with hematoxylin and eosin (H&E) and examined under a light microscope (Axioplan 2; Carl Zeiss Microscopy). Digital images were obtained with a microscope camera (Axiocam 208 color; Carl Zeiss Microscopy) using ZEISS ZEN Blue 3.5 software (Carl Zeiss Microscopy). Remaining sections of the lesion were selected for fluorescence immunohistochemistry (IHC).

Prior to IHC, the sections underwent deparaffinization and antigen retrieval at pH 6 (Dako EnVision FLEX Target Retrieval Solution, Low pH; Agilent Technologies, Santa Clara, CA, USA) in an oven set at 80°C for 1.5 hours. Anti-GFAP (Agilent Technologies) was used as a macroglial cell marker, identifying both astrocytes and Müller glia. Anti-GS (Sigma-Aldrich, St. Louis, MO, USA) was used to identify Müller glia, and anti–S100 calcium binding protein B (S100B; Sigma-Aldrich) was used to identify mainly astrocytes, as earlier described.^22^ Anti–proteinkinase C alpha (PKCα; Sigma-Aldrich) was used to identify rod bipolar cells and their processes.^26^^,^^27^ Dopaminergic amacrine cells were identified using an antibody recognizing anti–tyrosine hydroxylase (TH; Sigma-Aldrich). Sections were blocked with 5% donkey serum (Jackson Immunoresearch, West Grove, PA, USA) and incubated with primary antibodies overnight at 4°C. The following day, sections were washed and incubated with secondary antibodies for 2 hours. Finally, the sections were washed and mounted with glycerol/water (1:1) and 4′,6-diamidino-2-phenylindole (DAPI; 1:1000). Micrographs were taken (20×) on an iMIC confocal microscope (Till Photonics, Gräfelfing, Germany), as described previously.^27^

## Results

### Retinal Imaging With OCT and CFP

Retinal imaging was performed after 2 weeks in seven eyes, and two pigs were euthanized. Four weeks postoperatively, seven eyes were examined before euthanasia. All retinal holes were closed after 4 weeks, regardless of basal hole size. The AM sheets remained visible and in place in the subretinal space in seven out of nine eyes. In the two cases where the AM had dislocated to the vitreous, a RD was present in relation to the unclosed hole at 2 weeks (see Supplementary Fig. S1), and both pigs were euthanized. The eye with the largest retinal hole, measuring 2934 µm, presented with a tractional RD in relation to the closed hole. Other postoperative complications included cataract (*n* = 3) due to accidental lesion to the posterior lens capsule.

**Figure 2.**
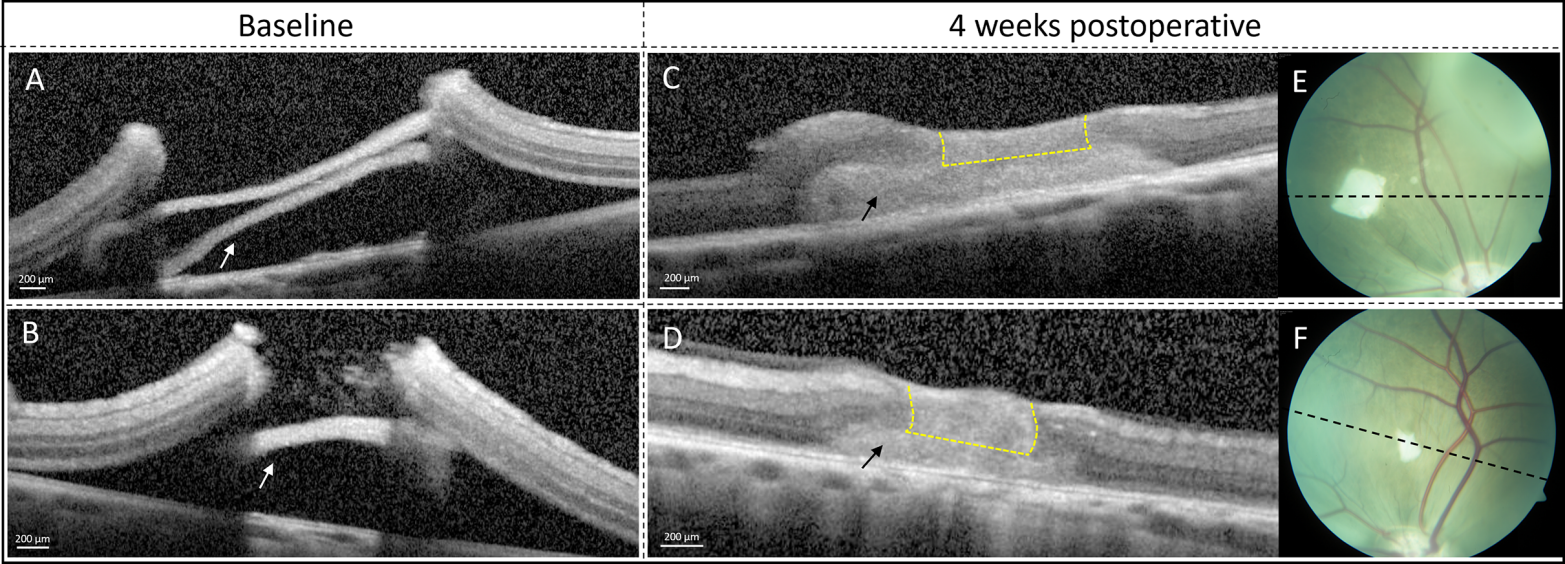
OCT and CFP immediately after surgery (baseline) and 4 weeks after surgery. (**A**, **B**) The AM sheet (*white arrows*) was placed in the subretinal space immediately after the retinal hole was established. (**C**, **D**) After 4 weeks, OCT scans showed hole closure with a gliotic plug (within the *yellow* *dashed lines*) above the AM sheet (*black arrows*). (**E**, **F**) CFP showed the AM sheet in place at the retinal hole site. The directional orientation of the OCT scan is indicated by the *black* *dashed* *lines*.

**Figure 3.**
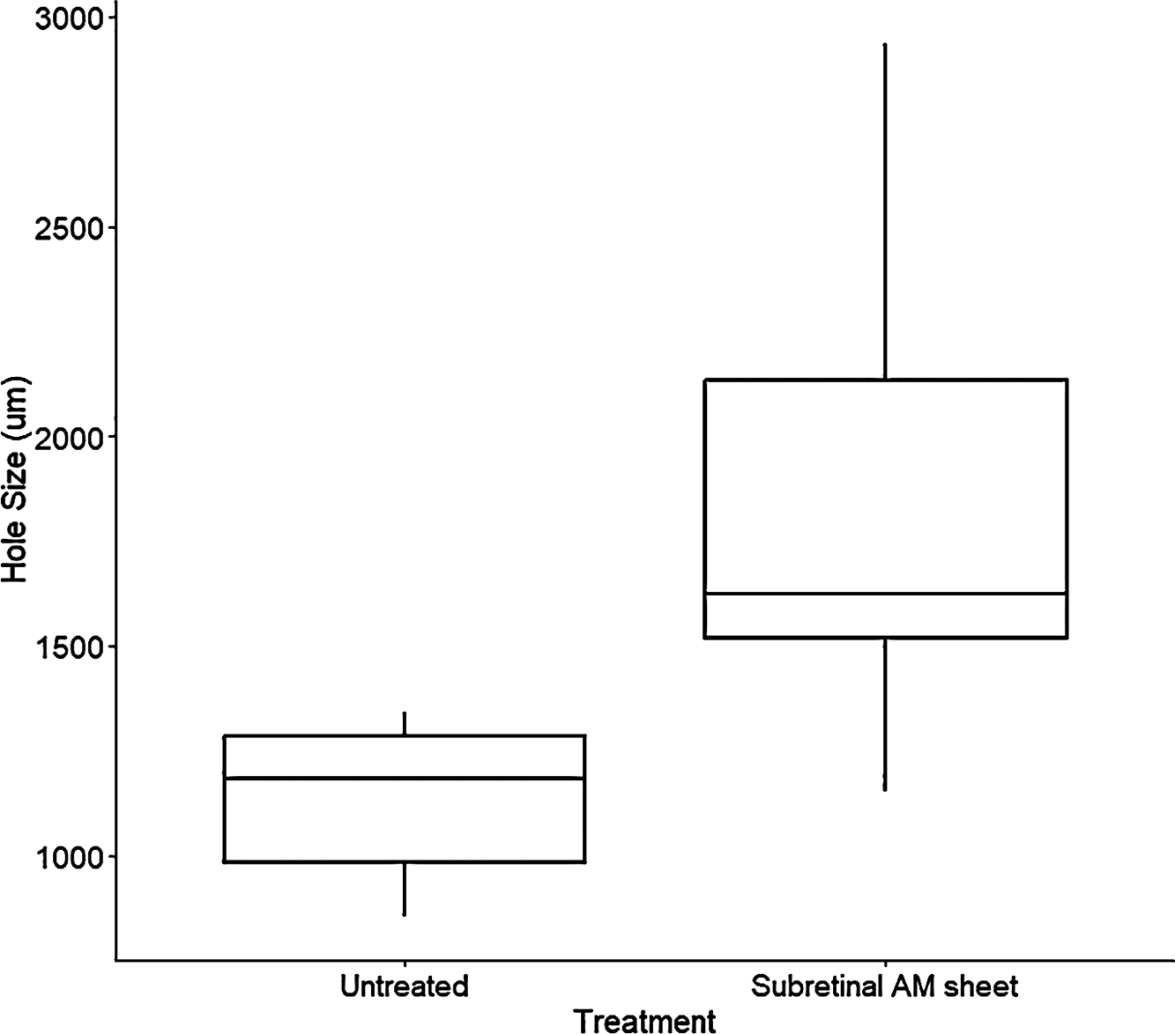
Closed retinal holes with and without amniotic membrane. The boxplot illustrates the basal dimensions of spontaneously closed holes (*n* = 5) and holes treated with a subretinal AM sheet (*n* = 5) (median, IQR, absolute range).

**Figure 4.**
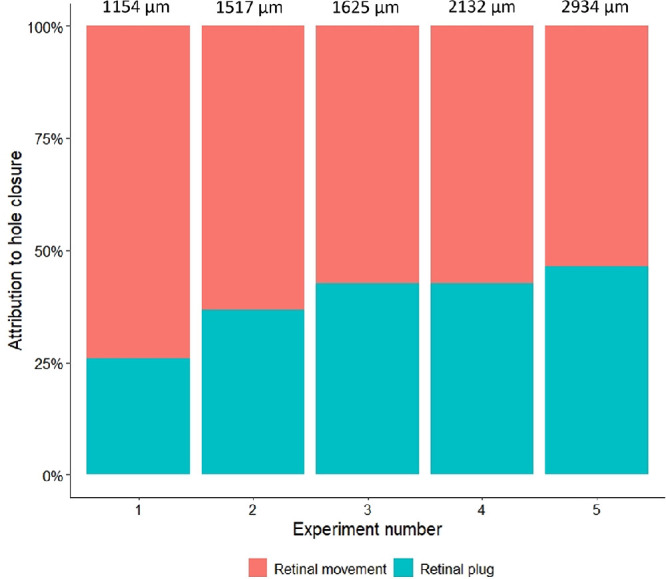
Hole closure mechanism following amniotic membrane transplantation. The experiment numbers correspond to the closed retinal holes at 4 weeks of follow-up arranged according to their size, smallest to largest. The mean hole size was 1872 µm. The bars depict the percentage of hole closure attributed to the retinal plug (*cyan*) and movement of the retinal hole borders (*red*). The percentage of the retinal plug increased with hole size.

Postoperative OCT demonstrated correct placement of the AM sheet in the subretinal space (Figs. 2A, 2B). The basal diameter of the hole was known in five out of seven eyes with a closed retinal hole. OCT imaging just after surgery was not possible in two eyes due to air tamponade. Follow-up OCT of the closed retinal holes showed a gliotic plug above the AM sheet between the original edges of the hole (Figs. 2C, 2D). The mean size of the holes that achieved closure 4 weeks after subretinal AM transplantation (*n* = 5) was 1872 µm (Fig. 4, calculated). In the untreated control group, the mean size of the spontaneously closed holes (*n* = 5) was 1130 µm. Basal hole sizes of the successfully closed holes with and without AM are illustrated in Figure 3. In the AM group, the median was 1625 µm (range, 1154–2934) with an interquartile range (IQR) of 615 µm. In the untreated group, the median was 1184 µm (range, 854–1341) with an IQR of 299 µm.

In all cases, the plug was much smaller than the basal hole. The difference between the basal hole and the retinal plug was attributed to the movement of the surrounding retina toward the center. Figure 4 illustrates the contribution to hole closure by the retinal plug and central retinal movement. Notably, the percentage of the retinal plug contribution to hole closure increased with the size of the hole.

**Figure 5.**
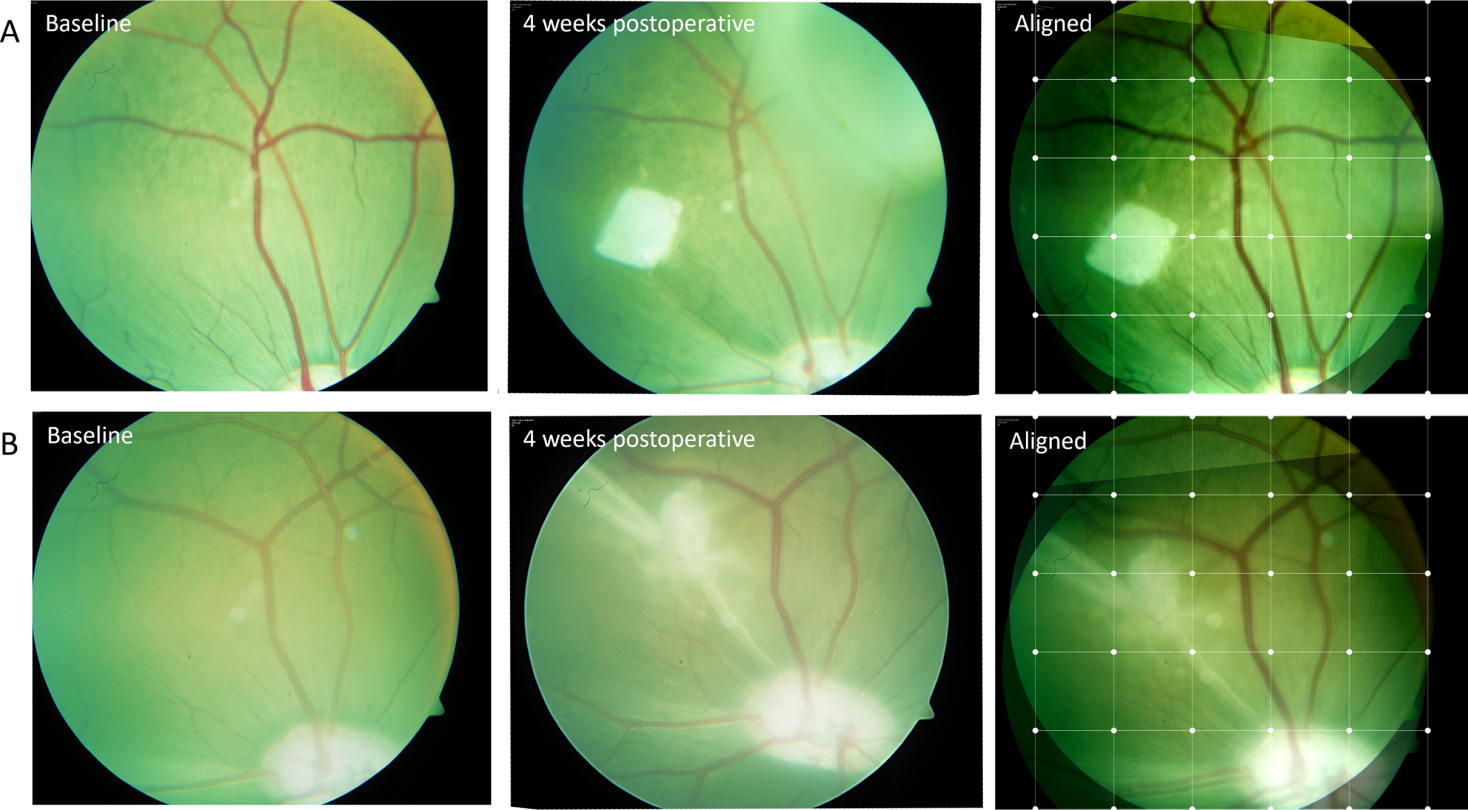
Movement of the retinal vessels in closed retinal holes. (**A**, **B**) Aligned baseline and 4-week follow-up fundus images of the two largest retinal holes in the study, measuring 2934 µm (**A**) and 2132 µm (**B**), respectively. In **A**, the aligned fundus images show duplication of the major retinal vessels, demonstrating induced vessel movement between the scan times. This case presented with a tractional RD. In **B**, the aligned fundus images show complete overlap of the major retinal vessels, indicating preserved vessel structure.

Assessment of retinal vessel movement was possible in six out of the seven eyes at the 4-week follow-up by using the Heidelberg OCT follow-up function. One eye was excluded, as cataract made it impossible to obtain an infrared image at follow-up. In one out of the six eyes, the reference image could not be retrieved at follow-up due to significant movement of the vessels, as illustrated in the aligned fundus images in Figure 5A. Notably, this was the case with the largest hole and a tractional RD. In the remaining cases, the follow-up sets were complete, indicating preserved vessel structure, as illustrated in fundal images of the second largest hole in Figure 5B.

### Histology

**Figure 6.**
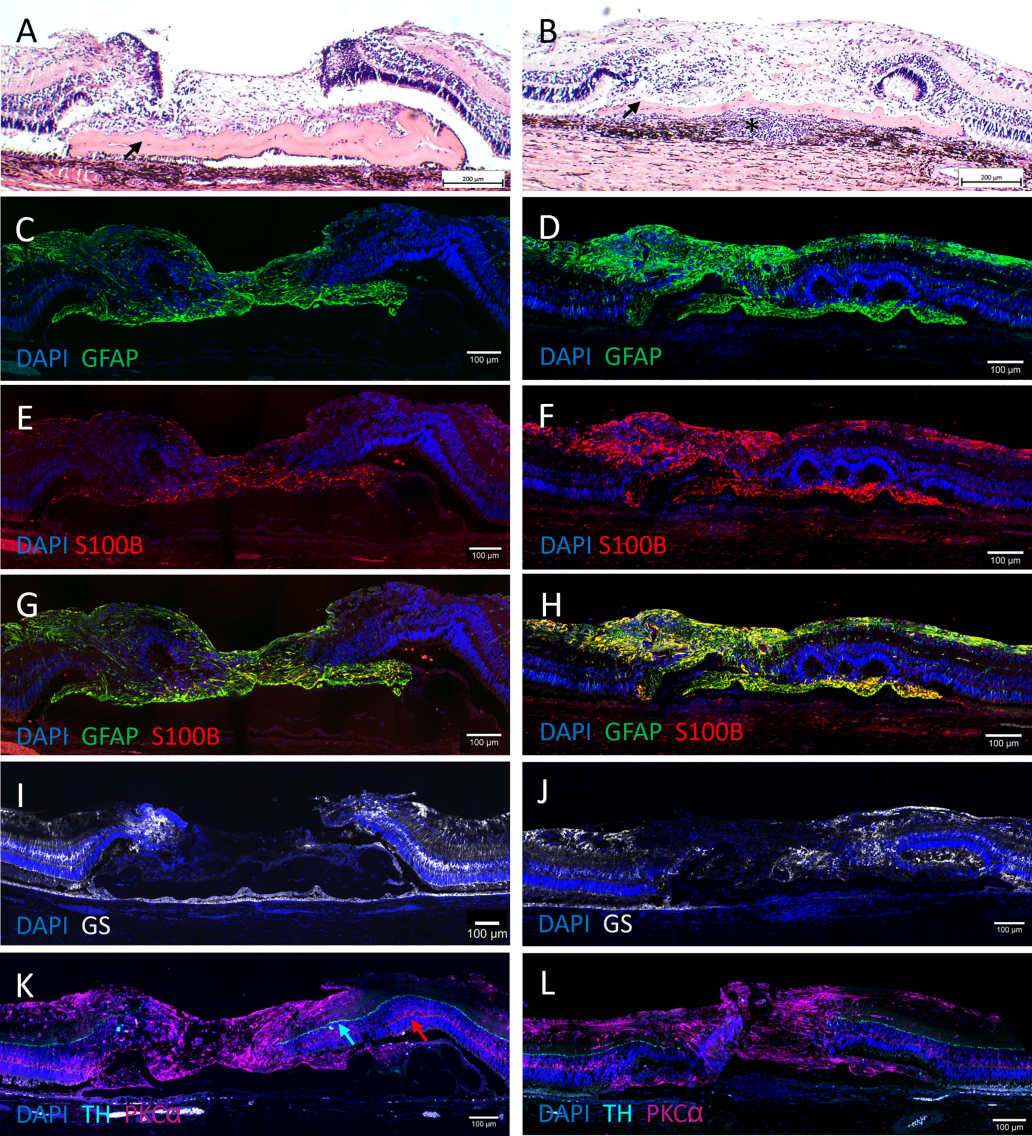
H&E staining (**A**, **B**) and IHC staining (**C**–**L**) at 4 weeks postoperative. (**A**, **B**) H&E staining micrographs (10×) of closed retinal holes showed a gliotic plug above the AM sheet (*black arrows*). The outer surface of the AM sheets was covered with RPE cells, and the inner surface was covered by gliotic tissue (**A**). Choroidal inflammation was observed in relation to a lesion in Bruch's membrane (*asterisk*) (**B**). (**C**–**H**) IHC staining micrographs (20×) showed that the plugs were positive for anti-GFAP (astrocytes and Müller glia) and S100B (mainly astrocytes). Merging showed increased colocalization in the plug (*yellow*). The AM sheet below the adjacent retina was also covered with glial cells. (**I**, **J**) Anti-GS (Müller glia) was negative in the plug. (**K**, **L**) Micrographs (20×) showed preserved retinal layers to the entirety of the margins of the hole. There was no invasion of amacrine cells, anti-TH (*cyan arrow*), into the plug. Sprouting of rod bipolar cells (*red arrow*) could not be excluded, as PKCα also stained positive within the plug.

We conducted histological analysis of the seven eyes that achieved closure within 4 weeks of follow-up (Fig. 1). H&E sections of the closed retinal holes showed that the gliotic plug disrupted the neuroretina, whereas the RPE remained intact beneath the AM sheet (Figs. 6A, 6B). The adjacent retina above the AM sheet was well preserved, with only minor damage to the outer segments (OSs) (Fig. 6A). In the subretinal space, the AM sheets displayed partial coverage by both RPE and gliotic tissue (Fig. 6A). The AM surface oriented toward the photoreceptor layer was predominantly covered by gliotic tissue, whereas the surface facing the RPE layer was covered with RPE cells. Choroidal inflammation was observed in four eyes in relation to a lesion in Bruch's membrane (Fig. 6B).

### Immunohistochemistry

IHC analysis revealed that the plug consisted of anti-GFAP–positive macroglial cells (Figs. 6C, 6D). More specifically, the plug stained positive for anti-S100B (Figs. 6E, 6F), which predominantly stains astrocytes. Enhanced colocalization was observed in the plug when anti-GFAP and anti-S100B were merged (Figs. 6G, 6H), whereas staining with the specific Müller glia marker, anti-GS, was negative (Figs. 6I, 6J). This finding suggests that astrocytes are the primary glial cells of the plug. There was no restoration of the structural retinal layers in the plug (Figs. 6K, 6L). When we stained with markers for inner retinal neurons (more specifically, amacrine and bipolar cells) using anti-TH and anti-PKCα, respectively, we observed that the retinal layers in most cases appeared preserved to the entirety of the margins of the hole (Fig. 6L). There was no invasion of amacrine cells across the borders of the hole to the plug (Figs. 6K, 6L). The plug stained positive for anti-PKCα. As anti-PKCα also labeled macroglial cells in the nerve fiber layer (NFL), the staining may primarily represent glial cells rather than sprouting rod bipolar cells (Fig. 6K).

## Discussion

In this study, we show that subretinal AM sheets promoted hole closure by gliosis and migration. Retinal holes treated with AM transplantation closed within 4 weeks of follow-up, regardless of their initial hole size. We have previously shown that untreated porcine retinal holes larger than 1380 µm remained open after 4 weeks with minimal sign of glial reaction at the hole margins, whereas smaller holes had closed spontaneously.^22^ In contrast, subretinal AM transplantation successfully closed retinal holes much larger than 1380 µm. The ability of AM to close large MHs in humans was recently noted by the CLOSE study group.^28^ They found superior efficacy of the AM and ART technique in cases with MHs larger than 800 µm, suggesting its potential as a first option in such cases.

The closure mechanism resembled that of spontaneously closed holes—namely, a centripetal migration of the margins of the edges of the hole and the formation of a gliotic plug.^22^ This mechanism is consistent with the concept underlying closure of MHs using the ILM flap technique in humans.^1^^,^^2^^,^^16^ In support of this, Shiode et al.^29^ recently demonstrated the presence of GFAP-positive macroglial cells in contact with and surrounding the ILM flap in a primate model of MHs.

Our IHC analyses indicated that astrocytes seemed to be the predominant glial cell of the plug. However, it is important to acknowledge the possibility that downregulated GS expression in Müller glia in response to gliosis^30^ adds uncertainty regarding their presence in the plug. It is important to clarify that our porcine model has several limitations in its comparison with MHs in humans, aside from pigs lacking a macula. The traumatic induction of a retinal hole itself may induce glial cell proliferation. Additionally, the young age of the included pigs may have led to a stronger healing response compared to a typical MH patient. Both factors may have affected the hole closure rate. However, given that spontaneously closed holes were much smaller than those closed with an AM sheet (Fig. 3), we postulate that hole closure following subretinal AM transplantation cannot solely be attributed to the healing response of the porcine retina. Thus, the AM sheet serves as a stimulator for glial proliferation and migration of glial cells to the retinal hole.

Our findings suggest that the time-dependent visual improvement observed in patients may be attributed to the initial anatomical closure of the hole with a gliotic plug, followed by gradual lateral movement of photoreceptors from the surrounding retina. During the 4 weeks of follow-up, we observed no structural restoration within the plug nor did we observe lateral invasion of cells into the plug. However, the surrounding retina that had migrated centrally to close the hole remained preserved above the AM sheet. Thus, the visual gain may be facilitated by the surrounding retina rather than actual retinal regeneration or migration of retinal neurons into the plug.

OCT imaging of patients who underwent subretinal hAM transplantations revealed a partial restoration of the outer retinal layers, more specifically the external limiting membrane and the ellipsoid zone during 6 months of follow-up.^7^^,^^10^ This may explain the continuous visual gain observed in patients during the initial 6 to 12 months following surgery,^7^^,^^10^^,^^12^ suggesting that retinal restoration may occur with time. It is important to acknowledge that we cannot completely reject the possibility of sprouting of rod bipolar cells to the plug. Although anti-PKCα is a well-known marker for rod bipolar cells, it has earlier been reported that some astrocytes and Müller glia in the porcine retina may exhibit positivity for anti-PKCα.^31^ This observation aligns with our findings, as we observed the presence of anti-PKCα–positive cells in both the NFL and the retinal plug. However, given the similarity in staining patterns between anti-PKCα and anti-GFAP in the retinal plug, it is more plausible that the observed staining represents anti-PKCα–positive glial cells. One major limitation of this study is the relatively short follow-up period, which prevented us from determining whether retinal remodeling occurs with time. Thus, conducting long-term animal studies with comprehensive histopathological analyses, such as in minipigs, would be valuable for future research.

Despite the closure of very large retinal holes, most cases did not exhibit observable retinal striae suggesting retinal contraction or tension around the closed hole. This observation suggests that the posterior pole of the porcine retina possesses a high degree of elasticity. Furthermore, we observed that the proportion of hole closure assigned to the retinal plug increased with hole size, suggesting a potential threshold for retinal stretching (Fig. 4). In our study, the primary mechanism of hole closure was attributed to the centripetal movement of the surrounding retina (Fig. 4). Although some of this movement can be assigned to the repositioning of the lifted hole margins to the RPE, we speculate that most of the retinal movement resulted from retinal stretching, given that tissue was removed to create the hole. Visible distortion of retinal vessels was only noted in one case with a tractional RD, which might solely explain the dragging of the vessels (Fig. 5). However, considering that this was also the largest hole in the study, it cannot be ruled out that the hole closure was contractive, as well. The absence of retinal vessel movement in the remaining cases supports the theory of retinal elasticity contributing to hole closure.^32^

Increased retinal mobility is indeed believed to be one of the primary reasons for the high hole closure rates following ILM peeling in standard MH surgery, as ILM removal reduces retinal rigidity.^33^^,^^34^ In our study, we did not perform ILM peeling; however, the local RD formed by the subretinal bleb may have enhanced retinal mobilization. Several patient studies have demonstrated that surgically induced macular detachment promotes closure of persistent or recurrent MHs.^35^^–^^39^ The rationale behind this procedure is the inherent elastic properties of the retina, facilitating convergence of the hole edges, together with the possibility that adhesions between the RPE and the edges of the MH may restrict MH closure.^39^ However, the key distinction between this method and our porcine retinal hole model is that we have removed retinal tissue to create the hole. The elastic properties of both the porcine and the human posterior eye wall have been evaluated earlier by Chen et al.,^40^^,^^41^ who found that the elastic moduli of each porcine tissue layer were comparable to those of human tissues, making the porcine eye an equivalent model for the human eye in relation to retinal elasticity.

The subretinal AM sheet exhibited a competitive ingrowth of RPE cells and glial cells. This is in accordance with previous observations indicating that RPE cell growth and gliosis happen in a competitive manner on subretinal basement membranes in the pig.^21^^,^^42^^,^^43^ Notably, the AM sheet beneath the surrounding retina was entirely covered with glial cells. There was minor damage to the OSs of the overlaying photoreceptors. However, this may be a temporary effect of the short-term retinal detachment, as earlier described.^44^ The presence of glial tissue in the subretinal space, which may influence the retinal function, suggests that the AM sheet should be cut as small as possible. Utilizing RPE-covered AM sheets instead of bare ones could potentially reduce the undesired gliosis observed beneath the surrounding retina. Furthermore, it may serve as a preserver of the adjacent photoreceptors. However, this strategy may impede glial cell migration to the hole, resulting in suboptimal closure. Alternatively, recent studies have investigated placement of the AM sheet on top rather than below the hole. This technique has proven effective for patients with complicated MHs associated with RD.^45^^–^^47^ Theoretically, the epimacular placement may better respect the structural retinal layeringand prevent the induction of gliosis between the MH edges, as seen when the AM is placed in the hole.^47^

Despite the creation of very large holes, RD associated with the retinal hole was only observed in eyes where the AM sheet had dislocated to the vitreous. The relatively high incidence of choroiditis may be attributed to either choroidal trauma or xenotransplantation-induced inflammation. Previous studies have documented choroidal responses following subretinal procedures in Landrace pigs. Lassota et al.^43^ reported localized choroidal inflammation due to the formation of surgical choroidal neovascularization membranes. In contrast, severe choroiditis was observed following subretinal xenografts of human neural progenitor cells in non-immunosuppressed Landrace pigs.^48^ Thus, we would expect a more pronounced choroidal inflammation if it were a consequence of xenotransplantation, indicating that the localized choroidal response is likely a result of choroidal trauma.

To improve maneuverability within the eye, the AM sheet was folded with the chorion facing inward. Consequently, we cannot ascertain whether a single-layer AM sheet with the chorion layer facing the RPE would have yielded different outcomes. The adhesive properties of the chorion may have contributed to fewer instances of dislocated AM sheets. Additionally, the AM sheet tended to curl up within the subretinal space over time. Both challenges could potentially be addressed by adding a rigid bioscaffold to the AM, thereby enhancing its maneuverability inside the eye and maintaining its smoothness in the subretinal space.

## Conclusions

Subretinal AM transplantation effectively closed all retinal holes within 4 weeks of follow-up, regardless of hole size. The AM sheet acted as a stimulator for hole closure by stimulating glial cell proliferation and served as a scaffold for centripetal migration of the hole edges*.* The AM sheet beneath the surrounding retina was covered with gliosis. No retinal restoration was observed within the plug.

## Supplementary Material

Supplement 1
