# Supplementary material for: Subretinal Amniotic Membrane Transplantation in a Porcine Model of Retinal Hole
Source: Invest Ophthalmol Vis Sci. 2024 Nov 25;65(13):52. doi: 10.1167/iovs.65.13.52 (PMC11601133; doi:10.1167/iovs.65.13.52)
Supplement: Supplement 1 [file iovs-65-13-52_s001.pdf]

Supplementary Figure S1

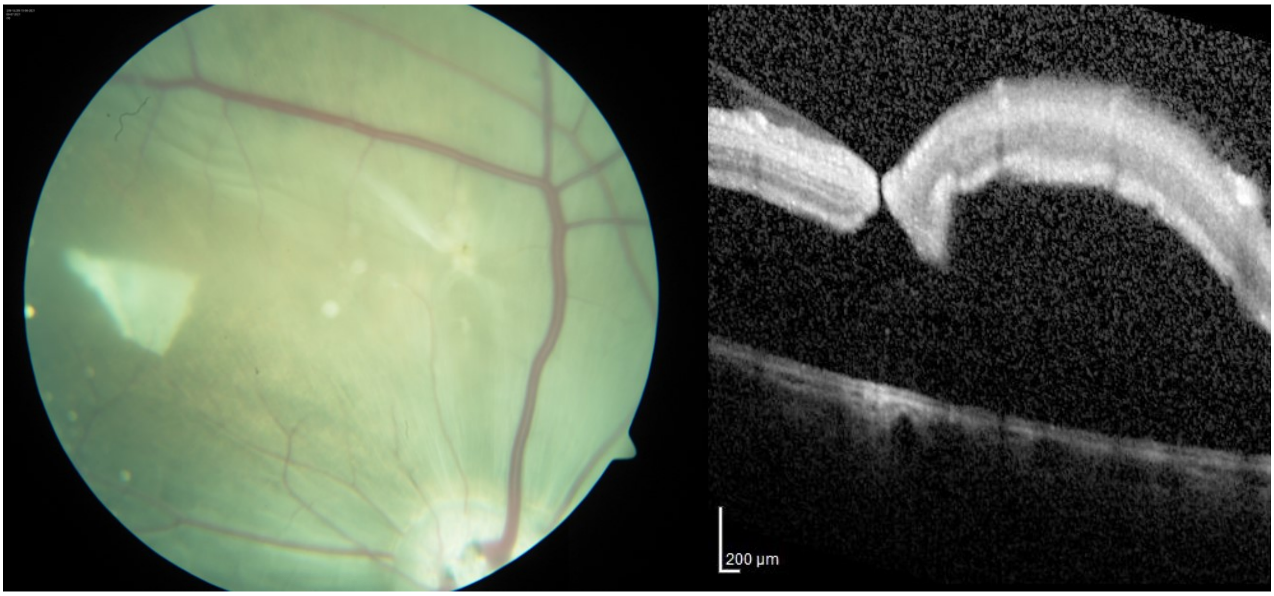

S1. Fundus photograph and optical coherence tomography show dislocation of the amniotic membrane sheet to the vitreous and a retinal detachment in relation to the retinal hole.
